# Effect of Propolis Paste and Mouthwash Formulation on Healing after Teeth Extraction in Periodontal Disease

**DOI:** 10.3390/plants10081603

**Published:** 2021-08-04

**Authors:** Maria Jesús Lisbona-González, Esther Muñoz-Soto, Cristina Lisbona-González, Marta Vallecillo-Rivas, Javier Diaz-Castro, Jorge Moreno-Fernandez

**Affiliations:** 1 Department of Stomatology, School of Dentistry, University of Granada, 18071 Granada, Spain; mjlisbona@hotmail.com (M.J.L.-G.); esther@msotodental.es (E.M.-S.); cristina.lisbona@gmail.com (C.L.-G.); mvallecillo@correo.ugr.es (M.V.-R.); 2 Department of Physiology, Faculty of Pharmacy, Campus Universitario de Cartuja, University of Granada, 18071 Granada, Spain; 3 Institute of Nutrition and Food Technology “José Mataix Verdú”, University of Granada, 18071 Granada, Spain; 4 Instituto de Investigación Biosanitaria IBS, 18016 Granada, Spain

**Keywords:** periodontal disease, propolis, chlorhexidine, teeth extraction, oral healing

## Abstract

This study investigated the antimicrobial effects of a mouthwash containing propolis and the effect of a propolis paste formulation on dental healing after teeth extraction in patients with periodontal disease. In the mouthwash experience, the population comprised 40 patients, which were divided as follows: the control mouthwash, 0.2% chlorhexidine (v/v) mouthwash, 2% (w/v) propolis mouthwash, and propolis + chlorhexidine mouthwash. The study of the propolis paste comprised a population of 60 patients with periodontal disease, and a total of 120 symmetric tooth extractions were performed. Propolis showed antimicrobial activity by itself, and especially with the chlorhexidine association. Three days after surgery in the teeth treated with control paste, only 13.4% had completely healed; however, with propolis paste, in 90% of the periodontal sockets, healing was complete. In addition, a reduction in *Streptococci mutans* and *Lactobacilli* cfu was observed with propolis, and especially with the association of chlorhexidine + propolis. Propolis mouthwash reduced bacterial proliferation, especially in association with chlorhexidine. Propolis paste is a viable alternative for socket healing after dental extraction. The knowledge gained from these findings will provide a foundation for similar propolis therapies in order to improve the healing process after dental surgery.

## 1. Introduction

Periodontal disease (PD) is a chronic infectious inflammatory disease that affects periodontium and gradually destroys the tooth-supporting alveolar bone.

The periodontium is a supporting structure that surrounds and supports the teeth. It consists of different tissues, including the gums, the cementum, the periodontal ligament, and the alveolar supporting bone. Periodontal diseases are caused by periodontopathic bacteria-derived factors and antigens that stimulate a local inflammation of gingival tissues resulting from dental-plaque-induced infection and activation of the innate immune system [1,2]. The characteristic tissue destruction results not from the pathogenic microorganisms, but from the host immune response; therefore, the aim of therapy is to attenuate neutrophil-mediated tissue injury and monocyte infiltration and restore periodontal tissue health [3].

Prevention of bacterial contamination of the operated area is essential to prevent wound infection and create a favorable environment for the healing process. The colonization of oral microorganisms and accumulation acids from bacterial metabolic activity, particularly *Streptococcus mutans* and other non-streptococcus species, such as *Lactobacillus acidophilus*, produce acid and bring the plaque to the critical pH. Plaque control is critical to maintaining good oral health, and it represents the gold standard to prevent periodontal health. If plaque formation is hindered, periodontal wound healing will be faster and with less complications.

The conventional treatment for periodontitis has focused on mechanical removal of bacterial agents, thus reducing infectious challenge and leading to resolution of inflammation and control of PD [4]. An alternative option was to enhance the regeneration of periodontal tissue through application of several substances, including the enamel matrix derivatives (Emdogain), antibiotics [5], and eritritiol [6]; however, these treatments did not significantly promote the healing of periodontal ligament cells [7].

In an attempt to improve this situation, propolis is a natural therapy that could be promising. Propolis, a resinous hive substance made by the honeybee, is a potent antimicrobial and anti-inflammatory agent because it comprises plant exudates and substances secreted in the course of bee metabolism. The main chemical classes present in propolis are flavonoids [8], phenolics [9], and various aromatic compounds. Flavonoids are well-known plant compounds that have antioxidant, antibacterial, antifungal, antiviral, and anti-inflammatory properties [10,11,12]. As an anti-inflammatory agent, propolis is shown to inhibit synthesis of prostaglandins [13], activate the thymus gland, aid the immune system by promoting phagocytic activity, stimulate cellular immunity, and augment healing effects on epithelial tissues [14,15,16,17,18]. Additionally, propolis contains elements, such as iron and zinc, that are important for the synthesis of collagen [19].

Bioflavonoids are known to help bleeding periodontal tissue and stimulate enzymes that fortify the walls of the blood vessels in the periodontium. This function of flavonoids is mainly attributed to their ability to inhibit prostaglandin synthesis and disinfect the tissue [20].

The purpose of this study was to evaluate the effect of a propolis-based medication supports the healing and epithelialization process after dental extraction. In addition, another experiment was performed to assess the effect of a propolis mouthwash on oral microbiota.

## 2. Results

### 2.1. Toothpaste Assay

**Figure 1:**
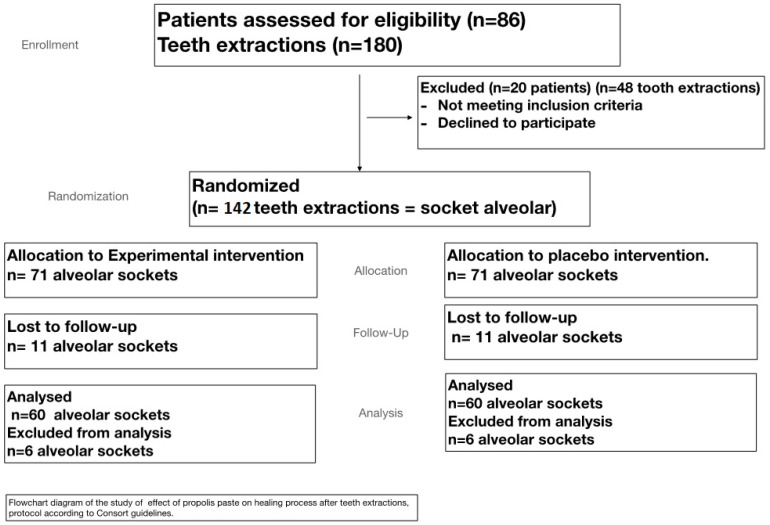
Flowchart of the toothpaste assay.

During the period available for the development of the study, a selection for convenience, in which 86 patients were eligible, was carried out. Because of the inclusion and exclusion criteria and availability, it was only possible to include a sample of 66 individuals. Finally, 60 subjects (28 males and 32 females) completed the period of study. One hundred and twenty extractions were performed, assigning them to the test or control group according to a randomization table. The study flow chart is shown in Figure 1.

During the first visit (3 days after surgery) in the socket treated with control paste, 8 dental sockets (13.4%) had completely healed (total closure of the socket), 34 sockets (53.4%) showed granulations, and 18 sockets (33.4%) showed partial fill in with soft gum tissue (partial closure). In contrast, with propolis paste, in 38 dental sockets (63.3%), periodontal healing was complete, 6 dental sockets (10%) showed granulations, and 16 dental socket (26.7%) showed partial closure. During the second visit (4 days after surgery) with control paste, 14 dental sockets (23.4%) had completely healed, 8 dental sockets (13.3%) showed granulations, and 38 dental sockets (63.3%) showed partial closure. In contrast, with propolis paste in 42 dental sockets (70%), periodontal healing was complete, and 18 dental socket (30%) showed incomplete/partial closure. Finally, in the third visit (7 days after surgery) with control paste, 36 dental sockets (53.4%) had completely healed, and 24 dental sockets (46.6%) showed partial closure. However, in all the dental sockets (100%) treated with propolis paste, periodontal healing was complete (Table 1).

### 2.2. Mouthwash Assay

**Figure 2:**
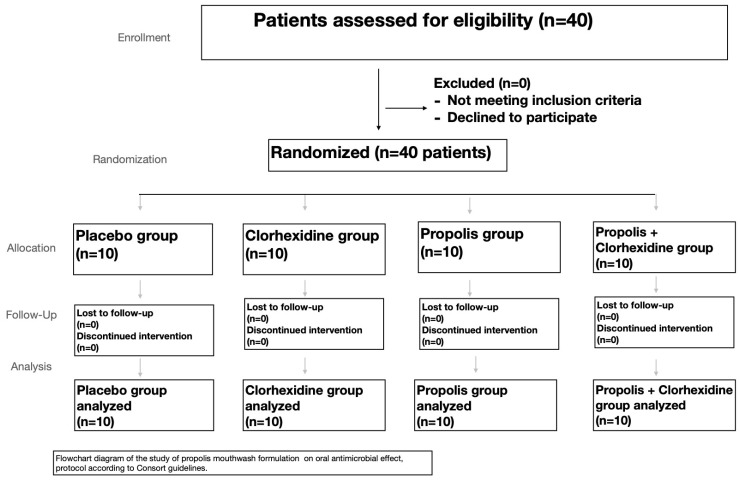
Flowchart of the mouthwash assay.

A subsequent recruitment of another 40 subjects for the mouthwash clinical experiment was carried out. (Figure 2). All 40 patients were randomly assigned to the different mouthwashes. The baseline scores of *Streptococci mutans* and *Lactobacillus* spp. were recorded after and before using the different mouthwashes.

The baseline level of *Streptococci mutans* score was 2 (colonies growth are >105 but <106 cfu), and *Lactobacilli* score was 3 (~105) (*n* = 40); 48 h after using the different mouthwashes three times per day, the results were as follows for *streptococcus mutans*: in patients using control mouthwash, score 2 in 100% of the patients; in patients using chlorhexidine mouthwash, score 2 in 20% of the patients, and 1 in 80% of the patients; in patients using propolis mouthwash: score 2 in 40% of the patients, and score 1 in 60% of the patients; in patients using chlorhexidine + propolis mouthwash: score 1 in 100% of the patients. With regard to the *Lactobacillus* spp., the results were: in patients using control mouthwash, score 3 in 100% of the patients; in patients using chlorhexidine mouthwash, score 2 in 100% of the patients; in patients using propolis mouthwash: score 2 in 50% of the patients, and score 3 in 50% of the patients; in patients using chlorhexidine + propolis mouthwash: score 1 in 100% of the patients. The values are shown in Table 2.

Chemical analysis revealed that propolis used in the current study contained 169.8 ± 4.1 mg GAE/100g as total phenolics and 32.1 ± 1.2 mg CE/100g as total flavonoids. The pH of the mouthwash formulations (data reported of three replications ± SEM) was 5.9 ± 0.72, 6.1 ± 0.55, 6.3 ± 0.44, 6.4 ± 0.31 for the placebo, the chlorhexidine mouthwash, the propolis, and the propolis + chlorhexidine mouthwash, respectively. The pH of propolis paste formulations (mean of three replications ± SEM) was 5.9 ± 0.48 and 6.0 ± 0.51 for the propolis paste and placebo formulation, respectively.

## 3. Discussion

After using the propolis mouth rinse, an oral examination conducted by the researcher revealed no lesion, and no inflammation was observed in the majority of the subjects; therefore, we conclude that none of the patients had susceptibility of allergy to the propolis. However, some of the patients reported a burning feeling in the oral mucosa for a short period of time when they used the chlorhexidine mouthwash. Some other patients reported that, during the period using chlorhexidine mouth rinse, they had a dryness sensation in the mouth and increased tartar. These side effects are common in chlorhexidine, and, to avoid interferences, these subjects were excluded from the study. Most of the patients reported tooth/tongue staining after using mouthwash. None of the patients reported any of these symptoms with the control or propolis mouth rinse.

The pH of propolis paste can be considered a nonirritant to the buccal in healthy people. The teeth socket treated with propolis showed no allergic reaction.

Another interesting observation was that the association of chlorhexidine and propolis avoided staining of the mouth/tongue typical in this type of mouthwash in all the patients.

Propolis has anti-inflammatory properties that speeds up the healing process and is widely used in folk remedies [10,12,21,22,23,24,25]. Our results agree with them; they are significant (*p* < 0.001) for healing at 3, 4, and 7 days after using propolis paste. These effects are associated to its chemical components, which vary depending on seasonal conditions [26,27,28,29,30]. According to Song et al. [23], caffeic acid is one of the compounds responsible for the anti-inflammatory action and acceleration of the healing of surgical wounds, reporting that this acid significantly inhibits the hydrolysis of arachidonic acid and the production of prostaglandin E2, as well as the release of histamine by mast cells in cell cultures. These factors are potent inflammatory mediators [31,32]. The anti-inflammatory effect of propolis has been preliminary assessed in the treatment of a variety of inflammatory and ulcerative conditions with low rates of minimal side effects [32,33,34]. The use of propolis for the treatment of mouth ulcers is a traditional therapy utilized by some communities in the Middle East. Samet et al. [35] reported that patients who took a 500 mg capsule of propolis daily supplement were shown to have a statistically significant decrease in the frequency of outbreaks of recurrent aphthous stomatitis. In addition, the effect in healing treatment may be also attributed to the presence of beeswax in the base, which was found to have anti-ulcer and anti-inflammatory effects, as stated in previous studies [11,36,37].

In the mouthwash clinical trial, a clear reduction in cfu was observed with chlorhexidine, propolis, and especially with the association of chlorhexidine + propolis, compared to the baseline scores Table 2. This finding is probably justified by the antibacterial and anti-inflammatory effects of propolis. The reduction of number of microorganisms in dental plaque resulted in decreasing of bulk. Some studies in vitro and in vivo are available in the scientific literature in which propolis, in several formulations, has demonstrated activity against periodontal pathogens [24,38,39,40,41,42,43]. The antimicrobial property of Brazilian propolis is attributed to the presence of flavonoids, phenolic acids, and their prenylated derivatives on its composition. Propolis has a complex chemical composition, considering the type of bee that produced it, the origin, and seasons of collection. Moreover, its action is dose time-dependent, and, in this study, we took into account the time of use, evaluation times, and the concentration of the mouth rinse. Some components present in propolis as flavonoids (quercetin, galangin, and pinocembrin), caffeic acid, benzoic acid, and cinnamic acid probably act on the microbial membrane or surface of the cell wall, causing structural and functional damage [44]. Synergistic effects of different compounds seem to be the most important process to explain the antibacterial activity of propolis, since it is well established that a single propolis component does not have an activity greater than the other components of propolis isolated [38]. As previously mentioned, propolis, to produce the anti-inflammatory effect, acts in the modulation of cytokines and inflammatory enzymes, such as the suppression of the production of prostaglandins, leukotrienes, and histamine [45,46]. Therefore, a reduction in the number of microorganisms in dental plaque results in the reduction of products released by them, which act as trigger of gingival inflammation, reducing the severity of plaque [47]. The results of the present study are similar to the study of Awawdeh et al. [48] and Kandaswamy et al. [49], who compared the antimicrobial activity of propolis with calcium hydroxide as intracanal medicament and determined that propolis was effective in eliminating the microorganism. In addition, it has been previously reported that propolis modulates the expression of antioxidative enzyme proteins, inducing a direct scavenge of free radicals, promoting DNA repair, and causing a reduction of the peroxides that can damage polyunsaturated fatty acids, preventing lipid peroxidation and tissue damage [50,51], a fact that can also explain the better healing process with the propolis paste. However, limitations of the study include: by limiting propolis, as with some other hive products, its composition varies with the flora of a given area, the time of collection, and the inclusion of wax contaminants, in addition the number of patients enrolled and the short duration of the study. Future clinical trials with a high number of patients and longer time periods will be necessary to elucidate the final effects of both formulations.

## 4. Materials and Methods

### 4.1. Study Protocol and Patients

The clinical trials were designed in accordance with the principles of the Declaration of Helsinki, following CONSORT guidelines [52] with approval by the Clinical Research Ethics Committee of the University of Granada (protocol number 819). Informed consent was obtained from all individual participants included in both studies.

**Figure 3:**
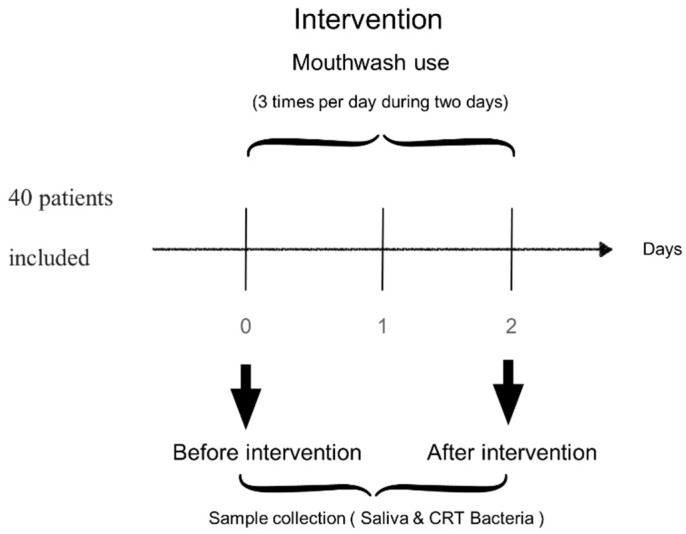
Study design. Visit protocol on mouthwash experience. The registered participants were 40 patients who had signed the informed consent and understood the procedure. They were randomly divided into four groups and were instructed to use mouthwash three times a day for two days. Sample of saliva and CRT bacteria were performed just before intervention, on the same day, and two days later.

A randomized, controlled clinical study was used in the mouthwash experience. Forty patients agreed to participate in the study, and 40 patients were screened for eligibility; they were in accordance with the inclusion and exclusion criteria and were then divided into four groups assigned randomly using a computer generated randomization (Table 2) *n* = 10 used the control mouthwash, *n* = 10 used the mouthwash containing 0.2% chlorhexidine (v/v), *n* = 10 used the propolis mouthwash containing 2% (w/v) of propolis, and *n* = 10 used the propolis + chlorhexidine mouthwash, containing 2% (w/v) of propolis and 0.2% chlorhexidine (v/v) (Figure 2 and Figure 3).

Patients were instructed to strictly follow oral hygiene instructions before entry into the study. The levels *Streptococci mutans* and *Lactobacilli* were measured before and after using the different mouthwashes, with commercial caries risk test, Vivacare line CRT (Caries Risk Test) bacteria 2 in 1 kit (Vivadent, Liechtenstein, Europe). The kit is comprised of a slide attached to the cover of the vial. The commercial product had one side of the slide coated with a solid selective culture medium (mitis salivarius agar enriched with sucrose) for the cultivation of *Streptococci mutans*, while the medium on the other side of the slide (Rogosa agar) was for the cultivation of lactobaccili. The salivary samples were used per the instructions of the manufacturer. The samples were incubated at 37 °C for 48 h. Growth density of the bacteria was evaluated under good lighting conditions by the naked eye and as per manufacturer’s instructions. Bacterial growth was then scored by comparing with standards expressed in colony forming units (cfu) provided by the manufacturers as follows: *Streptococci mutans* Scoring (0 = Very low colonies are detected; 1 = Low, colonies growth are <105 cfu; 2 = Medium, colonies growth are >105 but <106 cfu; 3 = High, colonies growth are >106 cfu) and *Lactobacilli* Scoring (0 = Very low colonies are detected; 1 = Low, colonies are ~<103 cfu; 2 = Medium, colonies are <104 cfu; 3 = High, colonies are >105 cfu). Raw propolis chunks scraped directly from the frames and boxes of bee hives were provided by Verbiotech I + D + i S.L. (Granada, Spain). Four alcohol-free mouthwashes were designed: the control mouthwash (placebo) containing glycerin, sodium benzoate, and purified water, the chlorhexidine mouthwash containing 0.2% chlorhexidine (v/v) added to the control solution, the propolis mouthwash containing 2% (w/v) of propolis, and the propolis + chlorhexidine mouthwash, containing 2% (w/v) of propolis and 0.2% chlorhexidine (v/v) added to the control solution. All the mouthwashes used in that study were prepared under aseptic conditions according to our request within the requirements of ISO 9001 and GMP International by Euronatur S.L. (Granada, Spain).

The second controlled clinical trial was designed following the same protocols, and it was approved by the same Ethics Committee (protocol number 819). Informed consent was obtained from all individual participants.

**Figure 4:**
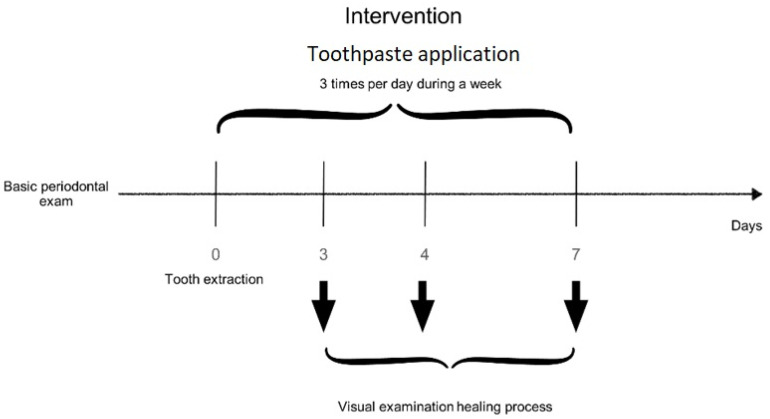
Study design. Visit protocol on healing process. The registered participants were 60 chronic periodontitis patients who had two or more teeth with high dental mobility, and they would need to have the extraction performed. Patients received test or control paste depending on randomization and were instructed to use it three times a day for one week on the dental socket after extraction.

Patients with chronic periodontitis were assessed for eligibility at the Department of Stomatology, School of Dentistry, University of Granada (Spain). Based on the guidelines for determining grade of periodontitis [53], in a total of 60 patients, a total of 120 symmetric tooth extractions were performed (Figure 1 and Figure 4). The follow-up period ranged during one week inclusion criteria were: patients giving informed consent, needing dental extraction with advanced periodontal disease, major bone loss, high dental mobility, and age between 50 and 60 years. Exclusion criteria were: lack of informed consent, systemic disease, allergies to any of the products tested, pregnancy or lactation, and use of antibiotics or anti-inflammatory drugs.

The propolis paste formulation was prepared under aseptic conditions. The required weight of dried components: pectin, carboxymethylcellulose, gelatin, methyl paraben, propyl paraben were mixed together to form a homogenous mixture. Beeswax was melted in water bath at 70–80 °C and continuously stirred with heating for 30 min. Subsequently, the homogenous mixture of the dried materials was gradually added to beeswax with continuous stirring and heating. Finally, the alcoholic extract of raw propolis (1 propolis: 3 ethanol) 10% w/w was added to the base gradually with continuous stirring till homogenous propolis paste was attained. The paste was poured into the collapsible tubes, closed properly, and stored in dry, cool place. Control paste formula (placebo) was prepared as the previously mentioned formula but free of the active constituent (alcoholic extract of propolis); both formulations used in the study were handled according to our request by Euronatur S.L. (Granada, Spain). For pH measurement, ten milliliters of each mouthwash were tested. One gram each of the paste formulations and the control was accurately weighed and dispersed in 10 mL of purified water. The pH of the dispersions and the mouthwashes were measured with a pH meter (Crison Instruments, Barcelona, Spain).

### 4.2. Propolis

Propolis samples were supplied by Verbiotech I + D + i S.L. (Granada, Spain). Its composition approximately contains 50% resin and vegetable balsam, 30% wax, 10% essential and aromatic oils, 5% pollen, and 5% other compounds [30].

### 4.3. Total Phenolic Assay

A freeze-dried sample of 0.5 g of raw propolis chunks was weighed and phenolic and flavonoid products were extracted with 50 mL 80% aqueous methanol on an ultrasonic bath Model 2510 EMS (Hatfield, PA, USA) for 20 min. An aliquot (1 mL) of the extracts was centrifuged at 14,000 rpm for 5 min. The total phenolic content of propolis and vegetable product was determined by the Folin-Ciocalteau assay [54]. The extracts were oxidized with Folin-Ciocalteu reagent, and the reaction was neutralized with sodium carbonate. The absorbance of the resulting blue color was measured at 760 nm after 60 min. Using gallic acid as standard, total phenolic content (standard curve was prepared using concentrations 2.5–50 mg/L) was expressed as mg gallic acid equivalents (GAE)/100g of fresh weight. Data are reported of three replications.

### 4.4. Total Flavonoid Assay

Total flavonoid content was measured by the aluminum chloride colorimetric [37]. An aliquot (1 mL) of extracts (0.5 g of propolis) was extracted in 50 mL 80% aqueous methanol), or standards solution of catechin (20, 40, 60, 80, 100 mg/L) was added to 10 mL volumetric flask containing 4 mL bidistilled H_2_O. To the flask, 0.3 mL 5% NaNO2 was added. After 5 min, 0.3 mL 10% AlCl3 was added. At the 6th min, 2 mL 1M NaOH solution was added, and the total volume was made up to 10 mL with bidistilled H_2_O. The solution was mixed well, and the absorbance was measured against prepared reagent blank at 510 nm. Total flavonoid content was expressed as mg catechin equivalents (CE)/100 g fresh mass. Samples were analyzed in triplicates.

### 4.5. Treatment Methods

Immediately after arrival of the patient in the department, the patients were diagnosed, and clinical and radiographic examinations were performed. A patient history was taken, which included duration and conditions of oral hygiene. The examination baseline consisted of a complete soft and hard tissues examination that was performed to register the condition of oral mucosa, so that any changes in the course of the study could be identified, making an assessment as to whether these changes could be related to the mouthwash. The mouth rinses were allocated according to groups to ensure balance. All subjects were instructed to rinse three times a day with the mouth rinse for one min and to refrain from all other oral hygiene measures until the final examination, two days later. The levels of *Streptococci mutans* and *Lactobacilli* were measured using commercial caries risk test, Vivacare line CRT (Caries Risk Test) bacteria 2 in 1 kit (Vivadent, Liechtenstein, Europe).

In the second clinical trial, exodontias will be assigned according to a randomization table carried out on the website www.random.org (accessed on 16 July 2021) to two groups (Test and Control), in which the only difference in protocolized action will be the topical application of an orabase paste added with 10% propolis, 2 times a day for 7 days on the socket alveolar after dental extraction (T), versus the application of orabase paste after dental extraction (placebo) (C). The investigator evaluating the postoperative period will be blinded for this assignment, while the patient and operator will not. Investigator and operator will be the same for all procedures. The same protocol was followed in all patients. Articaine anesthesia at 4% with adrenaline 1:100,000 (Laboratorios Normon; Tres Cantos, Madrid, Spain) and a simple dental extraction with drift and forceps. The patients did not receive antibiotic or anti-inflammatory treatment. The patients brought the amount of leftover gel to the check-ups to confirm its application.

### 4.6. Follow-Up Examination

All examinations were conducted by a single examiner trained to optimize the consistency of the study. Prior to the study, the adviser trained the dental examiner, as a “gold standard”, directing him to introduce the periodontal probe, gently, into the gingival sulcus, keeping the instrument parallel to the long axis of the tooth, and sliding it from the distal to the mesial so delicately in the buccal and lingual surface of each evaluated tooth. Eighteen dental extractions were performed prior to the study in ten patients to ensure the consistency of the method. The following information from the patient documentation (dental records and radiographs from treating dentists) was analyzed: type and number of injured teeth, age of the patients, and occurrence of complications. The clinical examination included inspection, palpation, periodontogram results, mobility grade, intraoral photographs, and radiographic examination of all teeth by using periapical bisecting angle exposures. Follow-up examinations were performed daily at two days into the mouthwash experiment. The patients used mouthwash three times per day, and they came back after 48 h to measure the bacterial concentration again according to protocol CRT bacteria^®^.

Follow up examinations in the wound healing study were at one week. During the postoperative week, the test group patients applied the 10% propolis paste added to the surgical wound three times a day for 7 days, and the control group patients applied the placebo paste) in the same way.

The healing variables measured at 3, 4, and 7 days will be the following, according to the modified table of Madrazo et al. [55].

### 4.7. Statistical Analysis

Since the survey comprised only 120 teeth in 60 patients and 4 mouthwashes in 40 patients, a comparative statistical analysis was performed with test “contrast of differences between proportions” (Chi-square test). Therefore, a descriptive analysis was performed using a frequency analysis, which is adequate for a survey sample of this size. A level of *p* < 0.05 was considered to indicate statistical significance. SPSS version 18.0, 2010 (SPSS Inc., Chicago, IL, USA), software was used for data treatment.

## 5. Conclusions

In summary, based on the obtained results, this study shows that the propolis mouthwash is effective in reducing the bacterial proliferation, especially in association with chlorhexidine, and with the added benefit that the association of propolis and chlorhexidine avoided the typical staining of the mouth/tongue when using this type of mouthwash. In addition, propolis paste is a viable alternative for socket healing after dental extraction and was more effective in controlling the inflammatory process over the experimental period. Therefore, the knowledge gained from these findings will provide a foundation for similar propolis therapies in order to improve the healing process after dental surgery.

## Figures and Tables

**Table 1:** Healing ratios and statistical significance of patients treated with control or propolis paste.

	Day 3			Day 4			Day 7		
	*n* (% Sockets)		*p*-Value	*n* (% Sockets)		*p*-Value	*n* (% Sockets)		*p*-Value
	Control	Test		Control	Test		Control	Test	
Completely healed	8 (13.3)	38 (63.3)	<0.001	14 (23.4)	42 (70.0)	<0.001	36 (60.0)	60 (100)	<0.001
Partial closure	18 (30.0)	16 (26.7)	0.096	38 (60.0)	18 (30.0)	<0.001	24 (40.0)	-	<0.001
Granulation tissue	34 (56.7)	6 (10.0)	<0.001	8 (13.3)	-	0.03	-	-	-

**Table 2:** Results of different mouthwashes’ formulation on oral antimicrobial effect.

Mouthwash	Baseline		48 h	
	Score (%)		Score (%)	
	*S. mutans*	*Lactobacillus* spp.	*S. mutans*	*Lactobacillus* spp.
Placebo *n* = 10	2 (100)	3 (100)	2 (100)	3 (100)
Clorhexidine 0.2% *n* = 10	2 (100)	3 (100)	2 (20) 1 (80)	2 (100)
Propolis 2% *n* = 10	2 (100)	3 (100)	2 (40) 1 (60)	2 (50) 3 (50)
Clorhexidina 0.2% + Propolis 2% *n* = 10	2 (100)	3 (100)	1 (100)	1 (100)

CRT^®^ bacteria (CRT^®^ Intro Pack—Caries Risk Test, Ivoclar Vivadent, Schaan, Liechtenstein). 1 = Low < 10^5^ cfu, 2 = Medium > 10^5^ < 10^6^ cfu, 3 = High *>* 10^6^ cfu.

## Data Availability

The data presented in this study are available on request from the corresponding authors.
